# Assessment of the Predictive Value of Spectrophotometric Skin Color Parameters and Environmental and Behavioral Factors in Estimating the Risk of Skin Cancer: A Case–Control Study

**DOI:** 10.3390/jcm11112969

**Published:** 2022-05-25

**Authors:** Marta Fijałkowska, Mateusz Koziej, Elżbieta Żądzińska, Bogusław Antoszewski, Aneta Sitek

**Affiliations:** 1 Department of Plastic, Reconstructive and Aesthetic Surgery, Medical University of Lodz, 90-419 Lodz, Poland; b.antoszewski@wp.pl; 2 Department of Anatomy, Jagiellonian University Medical College, 31-008 Cracow, Poland; mateusz.koziej@gmail.com; 3 Department of Anthropology, Faculty of Biology and Environmental Protection, University of Lodz, 90-136 Lodz, Poland; elzbieta.zadzinska@biol.uni.lodz.pl (E.Ż.); aneta.sitek@biol.uni.lodz.pl (A.S.)

**Keywords:** skin cancer, spectrophotometry, predictive factor, predictive model

## Abstract

Keratinocyte carcinomas are the most common cancers with different etiological risk factors. The aim of this study was to assess the predictive value of spectrophotometric parameters of skin color in correlation with environmental/behavioral factors to estimate the risk of skin cancer. The case–control study involved 389 patients. The analysis was performed on the training group to build a predictive model and on the testing group to check the quality of the designed model. Area under the curve based on the spectrophotometric skin parameters varied from 0.536 to 0.674. A statistically significant improvement of the area under curve was achieved by adding the number of sunburns for some models. The best single spectrophotometric measurement for estimating skin cancer is the skin melanin index measured on the arm or buttock. Spectrophotometric skin parameters are not very strong but are essential elements of models for estimating the risk of skin cancer. The most important environmental/behavioral factor seems to be the number of sunburns, but not the total exposure to ultraviolet radiation or usage of photoprotectors. Some other pigmentation predictors should be taken into account when creating new models, especially those that can be easily measured in objective and repeatable way. Spectrophotometric measurements can be employed as quick screening skin examination method.

## 1. Introduction

According to the latest terminology, non-melanoma skin cancers (NMSC) are now named keratinocyte carcinomas (KCs). They include basal cell carcinoma (BCC) and squamous cell carcinoma (SCC) [1,2]. Both neoplasms are derived from epidermal keratinocytes but diverge along distinct oncogenic pathways, leading to two phenotypically distinct cancers [3]. KCs account for 95% of malignant skin tumors [4]. Melanoma of the skin comprises only 1% of all skin cancers but it is responsible for approximately 90% of deaths associated with cutaneous tumors [4].

Keratinocyte carcinomas are the most frequently diagnosed neoplasms in the Western world, with one in every three tumors diagnosed as skin cancer [2,5]. Although the exact worldwide incidence of KCs is unknown, they represent a significant health burden in many countries [2]. As KCs mainly affects older individuals, and as the aging population in the world grows with increasingly longer life expectancies, BCC and SCC will continue to afflict more people worldwide [1,2,4].

Both genetic and environmental factors play a role in KCs and melanoma etiopathology. Susceptibility to skin cancers has been identified through family and familiar studies and genome-wide association studies [1]. The epidermis is exposed to many external environmental factors, including ultraviolet radiation, chemical agents (insecticides, fungicides, herbicides, and arsenic), and ionizing radiation [1,2,5,6]. These factors can cause mutations that eventually lead to carcinogenesis [1]. Since genetic factors are not modifiable, we should put our effort into changing alterable variables, such as sun exposure and sun-protective behaviors, to lower the risk of skin cancer formation. That is why it is important to know which behaviors are the most significant and should be eliminated from the healthier lifestyle.

Some medical models identify individuals at the highest risk for KCs or melanoma [2,7,8]. However, presented models vary—different factors are included, quite a small sample of patients are described—so they cannot be generalized to the worldwide population. However, such a screening tool is needed to diagnose skin cancer at an early stage or even prevent its formation from reducing morbidity and mortality in an aging global society and associating costs.

One of possible method that can be used to estimate skin color is spectrophotometry, which offers a suitable, objective, and reproducible way for evaluating skin pigmentation [9]. Measuring the optical attributes of the skin depends on the degree to which the skin reflects, absorbs, diffuses, and transmits incident light. The surface of the skin is heterogeneous, and depending on localization, the skin has different blood supply and pigmentation. Most of the pigment is found in the basal part of the epidermis. In the dermis, blood (mainly hemoglobin), beta-carotene, and bilirubin can absorb light, while the fibrous structure of the dermis can diffuse light. Skin erythema causes reddening of the skin and is associated with dilatation of blood vessels closest to the skin’s surface. The spectrophotometric analysis of the skin under controlled lighting conditions provided by the spectrophotometer (with white light emitting diode-LED) offer a precise characteristic of individual pigments. In clinical practice, spectroscopy is routinely used to assess the level of melanin (melanin index (MI)—the higher is the index value, the darker the skin) and level of erythema (erythema index (EI)—the higher is the index value, the stronger the skin erythema). In addition to melanin and erythema measurements, the most currently used spectrophotometers provide additional skin color measurements in the RGB system (R: red; G: green; B: blue; values range from 0 to 255, 0 for all of the coordinates gives black color, whereas 255 is white) and the CieLab color space (three coordinates: L, lightness—values range from 0 (black) to 100 (white); a, the green–red axis–positive values indicate the amount of red whereas negative values indicate the amount of green; b, the blue–yellow axis—yellow is positive and blue is negative).

This study aimed to assess the predictive quality of the spectrophotometric parameters of human skin color proposed in the previous work as variables useful for predicting the risk of skin cancer [10] and chosen environmental and behavioral factors to build the most precise prognostic tool.

## 2. Materials and Methods

**Figure 1:**
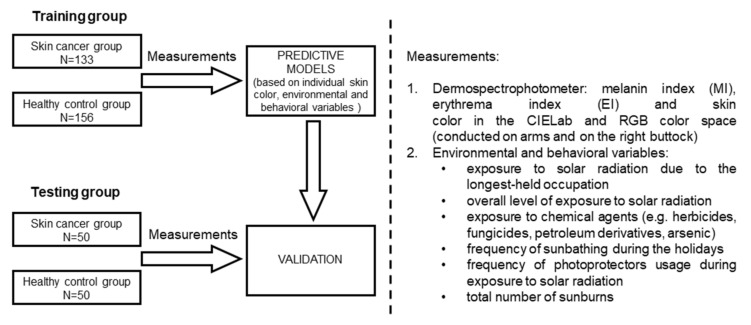
Graphic abstract to summarize the methodology. MI—melanin index; EI—erythema index.

The study material was based on two groups: the training group and the testing group. The testing group validated the models created on the basis of the training group (Figure 1).

The training group was examined between 2011 and 2014 and consisted of 289 people of Polish ancestry. There were 133 patients with skin neoplasms (100 with BCC, 21 with SCC, and 12 with melanoma) aged from 41 to 92 years and 156 healthy controls aged from 45 to 93 years (Table 1). The testing group was examined between 2020 and 2021 and consisted of 100 people of Polish ancestry. There were 50 patients with skin neoplasms (46 with BCC and 4 with SCC) aged from 43 to 90 years and 50 healthy controls aged from 45 to 87 years (Table 1). Written consent to participate in the study was provided by all patients. The study was approved by the ethics review board of the University of Lodz (approval no. KBBN-UŁ/II/8/2010) and the Medical University of Lodz (approval no. RNN/364/18/KE).

The inclusion criteria were as follows: no diseases related to skin pigmentation, no exposure to ultraviolet radiation (holidays, indoor tanning) in the last 3 months before the test, no usage of skin bronzing cosmetics, age above 40 years. All patients (both from the training group and testing group) were treated at the Plastic, Reconstructive and Aesthetic Surgery Clinic of the Medical University of Lodz. In all patients, the diagnosis was confirmed by histopathological examination. People from control groups were treated for scars, posttraumatic deformities, eyelid ptosis, xanthelasma, or chronic wounds.

The same study plan was used for both trials. Therefore, the same dataset was available for all respondents. Because in the training group the number of patients with SCC and melanoma was not numerous, the binary system was introduced; finally, two groups were analyzed: patients with skin cancers (regardless of type) and people without skin neoplasm. The same procedure was applied in the testing group.

### 2.1. Study Plan

The same dermospectrophotometer was used to measure the training and testing group. The skin was tested by DSM II equipment (Cortex Technology, Hadsund, Denmark). The following measurements were performed: melanin index (MI), erythema index (EI), and skin color in the CIELab and RGB color space (as mentioned in the Introduction section). Skin color measurements were conducted on the medial regions of the right and left arms and on the right buttock. The measurements were conducted in triplicate in each region, each time at a slightly different location, avoiding melanocytic moles and visible discolorations. Eventually, the mean value from three measurements from the right buttock and the mean value from six measurements from arms (three from right one and three form left one) were included in the statistical analysis.

The set of environmental/behavioral variables was selected a priori on the basis of the literature. The most common six variables were chosen: (1) exposure to solar radiation due to the longest-held occupation; (2) overall level of exposure to solar radiation; (3) exposure to chemical agents (e.g., herbicides, fungicides, petroleum derivatives, arsenic); (4) frequency of sunbathing during the holidays; (5) frequency of photoprotectors usage during exposure to solar radiation; (6) total number of sunburns (the number was given by the each patient). The first three factors were assessed in the scale from 1—no exposure to 5—very high exposure. The possible answers to the points concerning frequency (factor 4 and 5) were never, rarely, sometimes, often, and always.

### 2.2. Predictive Models

Logistic regression was used to develop the predictive models. Models were built on the basis of individual skin color variables (MI, EI, R, G, B, L, *a*, *b*) as well as on sets of variables within various skin color evaluation systems (MI and EI, CIEL*ab*, RGB). These spectrophotometric parameters that were selected as best predictors by Sitek et al. were used to build up models in the current study to validate them. Six models based on the parameters of arms skin color (MI, R, MI with EI, L*ab*, L*a*, L*b*) and five models based on the parameters of the buttock skin color (MI, R, MI with EI, L*ab*, L*a*) were chosen as the best predictors. In the next step, these models were supplemented with such environmental/behavioral variables that showed statistically significant relationship with skin cancer. Models were built on the training group, and their quality was assessed on the testing group (validation process).

However, the age and sex were not included as predictors in assessed models. The age was not taken into account due to the fact that some people from the testing group were not able to provide the exact time of appearance of the skin lesion. Thus, we could not determine the age of onset of the disease (assuming that the lesion was malignant from the beginning). The age at the study point also could not be used as a predictor because the time between the appearance of the lesion and the patient’s first visit may take up to several years. Additionally, what was shown in previous research was that the age of the subjects did not affect the prediction results in any of the models, and therefore it was not relevant in connection to spectrophotometric measurements as they can be independent variables [10]. In addition, in both studies, the inclusion criterion was the patient’s age at the time of the study—at least 40 years, which excluded younger patients and thus disturbed the probabilistic nature of the trial. The sex was also not taken into account due to the fact that the study was based on patients who themselves came to the Department of Plastic Surgery for consultation/treatment due to having some distressing skin lesions or for other reasons. In both groups (training and testing), the majority of patients were females, probably due to the fact that women consult doctors more often about their health problems than men. Therefore, none of the samples (training and testing) can be considered as representative in terms of age and sex.

### 2.3. Statistical Analysis

To compare categorical data for the sex, the chi^2^ with Yates correction was used. The Pearson chi^2^ test was used to compare the distribution of cancer types in groups. The Mann–Whitney test was used to check the difference between ages (Z-corrected).

Logistic regression was used to assess the influence of particular environmental and behavioral factors on cancer occurrence, followed by standard models and stepwise regression models to obtain an optimal set of parameters to assess the risk of skin cancer.

The predictive quality of particular models was assessed by receiver operating characteristic (ROC) curve and area under the curve (AUC) for the tested group (validated group). The AUC was within the range (0–1). However, values higher than 0.5 indicated that the analyzed factor had a useful predictive value. The higher AUC, the better the predictive value. The Hanley proposed algorithm for the Z model can be used to compare two AUC values [11]. The Holm–Bonferroni corrections were used for multiple comparisons. Statistical analyses were performed using StatSoft STATISTICA 13.1 software for Windows (StatSoft, Tulsa, OK, USA).

## 3. Results

### 3.1. Demographic Characteristic of Training and Testing Groups

Training and testing groups have not presented significant differences for sex and occurrence of skin cancer. There was a statistically higher frequency of BCC in the testing group than the training group, and a higher percentage of SCC in the training group than the testing group. MM was not represented in the validation group. There was a significant difference between ages; participants in the training group were older than the testing group, although the age range was similar (more than 40 and less than 95 years) (Table 1).

### 3.2. The Predictive Quality of Models According to the Spectrophotometric Parameters of Skin Color to Estimate the Risk of Skin Cancer

The parameters for spectrophotometric models were created on the basis of training data (Supplementary Data, Table S1). On the basis of the testing group, it has been shown that AUC for analyzed models varied from 0.536 (L, *a*, b of buttock skin) to 0.674 (MI for the inner side of the arm). The Z-test demonstrated that R parameter (of RGB system) for arm and buttock skin, as well as coordinates L, *a*, *b* and L, *a* for buttock skin, were not useful in predicting the risk of skin cancer because AUC for these variables was not significantly different than 0.5. This means that these parameters did not provide a better prediction than the random assignment of people to the patient group or healthy group. The remaining variables described skin color increase in the ability to predict the disease (Table 2). The model with the highest AUC (0.674) was based on the MI indicator for the skin of the arm and was not demonstrated as a significantly better predictor from remaining spectrophotometric parameters with AUC > 0.5 (Tables S2 and S3).

### 3.3. The Predictive Quality of Spectrophotometric Models Extended with Environmental and Behavioral Factors Significantly Associated with the Risk of Skin Cancer

The univariate logistic regression analyses revealed that out of the six variables mentioned in the Materials and Methods section and in the graphical abstract, only three showed a relationship with skin cancer: (1) exposure to solar radiation related to the longest-held occupation (Wald statistic = 4.47, *p* = 0.0342), (2) frequency of using photoprotectors (never vs. ever) (Wald statistic = 4.55, *p* = 0.0330), and (3) the total number of sunburns (Wald statistic = 10.86, *p* = 0.0010). In the next step, spectrophotometric models were completed with these factors, and by use of the testing group, the predictive utility of the extended model was assessed (checked if AUC was different than 0.5). Then, they were compared with initial spectrophotometric models. The parameters of the extended models with the three environmental/behavioral variables mentioned above are presented in Table S2. It is worth noting that after including all three environmental/behavioral variables in the spectrophotometric models, two of them (exposure to solar radiation associated with the longest occupation and the frequency of using photoprotectors) lost their significance in each classifier (Table S2).

When three environmental/behavioral variables were added, the AUC significantly exceeded 0.5 for the coordinate R models for the arm and buttock skin, which were not significantly different from 0.5 before modification. However, this extension did not result in the predictive utility of models based on the L, *a*, *b* and L, *a* coordinates and for the buttock skin (Table 2).

As a result of comparing the initial and extended models, no statistically significant differences were found in terms of their classification quality (Table 3). The modified model with the highest AUC (AUC = 0.689), which included, apart from the MI for the skin of the arms, three environmental/behavioral variables after taking into account the Holm–Bonferroni correction, did not turn out to be a statistically significantly better classifier than the remaining extended spectrophotometric models with AUC > 0.5.

### 3.4. The Predictive Quality of Spectrophotometric Models Extended with the Number of Sunburns

Statistical model building by backward stepwise method included the gradual removal of consecutive environmental/behavioral variables from the extended model until only one significant factor remained in the model. It turned out that in each case, the only expansion variable remaining in the model was the number of sunburns. This factor significantly increased the AUC of the three spectrophotometric models (Table 4, Table 5 and Table S4). The highest value of AUC was revealed for the model based on L, *a* parameters for the skin of arms (AUC increased from 0.650 to 0.691, *p* = 0.0160) (Table S3).

In the next stage of the analysis, it was checked as to whether the extended model with the highest AUC = 0.691 (L, *a* for the skin of arms, the number of sunburns) is a statistically significantly better classifier than the other models increased by the number of sunburns. After introducing Holm–Bonferroni correction, the Z test did not show any differences in terms of this (Table 4, Table 5 and Table S5).

## 4. Discussion

Skin cancers have become a serious worldwide health problem, and their incidence is increasing [12]. This is why it is important to emphasize early detection and treatment of skin neoplasms to decrease the morbidity, mortality, and costs associated with treating the tumors, especially in their advanced stages [13]. Setting up a tool that can assess the risk of skin cancer formation requires objective methods that can be measured in an independent, reliable, and honest way.

In the current study, we used objective method spectrophotometry to build a model for estimating the risk of skin cancer. We found that the predictive value of the following spectrophotometric classifiers: MI for arms and buttock skin and L, *a*, *b*, L, *a*, L, *b* for arms skin, confirms our previous results. The AUC values for these variables in both studies ranged from 0.6 to 0.7. This means that they were not strong classifiers but had a valuable contribution in terms of estimating the risk of skin cancer. Of course, they are not a sufficient tool alone, but they can fulfill their function when enriched with other predictors.

Additionally, we found that for some spectrophotometric skin color parameters (L, *a* for arms and buttock skin and R for buttock skin), the prediction quality is improved when information on the number of sunburns is included. At the same time, the number of sunburns in these models successfully replaced the information about the level of sun exposure and the frequency of photoprotectors usage. This is a valuable message as information about the number of sunburns is quite easy to obtain from the patients (they usually remember that they had blisters on the skin after sunbathing) while assessing the total exposure of sun (from no exposure to very high exposure), and the frequency of photoprotector usage (never, rarely, sometimes, often, always) is very subjective and depends on the patients’ opinion, so the bias cannot be excluded.

After adding the information on the number of sunburns, the highest classification quality was achieved by the parameters L, *a* for arms skin (AUC = 0.691), but this value did not differ, among others, on the predictive quality of melanin index for arms (AUC = 0.686) and buttocks (AUC = 0.665) extended by the number of sunburns, as well as the predictive quality for the melanin index itself for arms (AUC = 0.674) and buttocks (AUC = 0.643). Due to the fact that there is no difference between mentioned spectrophotometric classifiers, we suggest using the simplest of them—the skin melanin index (MI).

The lack of influence of environmental and behavioral variables (the level of sun exposure and the frequency of photoprotectors usage) on the predictive quality of melanin index (MI) may result from the fact that this index is directly related to the melanin level, which determines the skin sensitivity to the sun, and thus it correlates with the number of sunburns, making these variables redundant to each other. Performing spectrophotometric skin measurements during routine patients’ visit to out-patient clinics may have clinical meaning in skin cancer prevention as the melanin index provides quite good predictive quality. The technique of measurements is easy and can be done by all doctors in contrast to, e.g., dermatoscopy, which requires a specialist knowledge and training.

Various risk prediction models of skin cancer formation have been described in the literature. Most of these refer to melanoma alone, and there are many different risk factors described with little consistency and poor validation in the model evaluation, as stated by Kaiser et al. [8]. Good prediction with high AUC was presented by Bhalla et al. [14]. The authors used genomics profiles of patients, and their results showed that AUC can be more than 0.93 when analyzing mRNA and 0.84 or more when analyzing miRNA [14]. However, advanced genetic studies are not possible in all populations, mainly due to the lack of such databases in general populations and their costs. In another study, it was shown that elevated concentrations of proteins such as cathelicidin and β-defensin 2 are associated with the presence of BCC [15]. However, these factors are not specific only to this condition, which is why it could be interesting to find a relationship between spectrophotometric skin measurements and the above-mentioned proteins.

Roffman et al. presented their model of predicting KCs via a multi-parameterized artificial neural network and reached AUC from 0.79 to 0.82 [16]. The described model was mainly based on demographic data with other comorbidities that patients suffer from [16]. A similar model extended with photosensitizing medicaments was proposed by Wang et al. with an even higher AUC from 0.87 to 0.91 [17]. However, even these authors emphasized that further clinical studies are needed and that models require validation efforts to prove their efficiency because models were based only on training groups [16,17]. According to the Newcastle Ottawa Scale, both of the above-mentioned studies were assessed as “poor” [18]. In the current study, we present the validated results. Usually, such results turn out to be worse than those generated on the training group, but it provides the true priority of designed model.

It is well known that skin color or skin phototype is related to skin neoplasm. Fitzpatrick proposes a descriptive scale; however, this is not popular due to its subjective manner. In one study, phototype III/IV was paradoxically associated with an increased risk of BCC compared with phototype I/II [19]. The descriptive method of characterizing skin color as light/fair or medium versus dark/olive is also confusing and subjective [20]. A previously presented study has shown that AUC for models predicting skin cancers based on Fitzpatrick’s phototypes is 0.576, and it is lower than AUC for models based on spectrophotometric parameters (AUC = 0.661–0.687). This results from the fact that dermal spectrophotometry, performed in this research, offers objective measurements, which remarkably increase repeatability and enable the exact, quantitative assessment of skin color.

This study is not free from limitations. This is mainly due to the small number of examined cases. However, they provide the validation of results, which many studies do not present. Another limitation is our placement of all types of cancers in one group. Further studies on separate skin cancer groups are needed to check spectrophotometric models in patients separated by the types of cutaneous neoplasm.

## 5. Conclusions

Spectrophotometric skin parameters are essential but not very strong predictors for estimating the risk of skin cancer. The simplest spectrophotometric predictor of skin cancer is the melanin index (similar predictive value, whether measured on the skin of the inner surface of the arms or the buttock), and it should be included in predictive models. From environmental/behavioral factors, the most important one seems to be the number of sunburns, but not the total exposure to ultraviolet radiation or usage of photoprotectors. Further studies concerning skin cancer predictive models based on spectrophotometric measurements should be continued, but perhaps some other pigmentation predictors should be taken into account (like iris and hair color) when creating new models. In future, spectrophotometric measurements can be employed as a quick screening skin examination method.

## Figures and Tables

**Table 1:** Characteristics of training and testing groups.

Characteristics		Training Group	Testing Group	Training Group vs. Testing Group
Evaluation time		2011–2014	2020–2021	
N		289 (100%)	100 (100%)	
Sex	F	189 (65%)	61 (61%)	χ^2^_Yates_ = 0.45 *p* = 0.5029
	M	100 (35%)	39 (39%)	
Absence of skin cancer		156 (54%)	50 (50%)	χ^2^_Yates_ = 0.33 *p* = 0.5680
Presence of skin cancer		133 (46%)	50 (50%)	
Type of skin cancer	BCC	100 (75%)	46 (92%)	χ^2^ = 7.41 *p* = 0.0246
	SCC	21 (16%)	4 (8%)	
	MM	12 (9%)	0 (0%)	
Age (years)		M = 69; Q_1–3_ = 62–78 min–max = 41–93	M = 67; Q_1–3_ = 56–75 min–max = 43–90	Z_correction_ = 2.37 *p* = 0.0179

M—median; BCC—basal cell carcinoma; SCC—squamous cell carcinoma; MM—melanoma malignum; Q_1–3_—lower and higher quartile; Z_correction_—testing statistics for Mann–Whitney test. *p* < 0.05—result statistically significant.

**Table 2:** The predictive quality of analyzed models validated on testing group.

Models	Spectrophotometric Parameters	I		II		III	
		AUC	SE	AUC	SE	AUC	SE
Arm							
1	MI	0.674	0.0549	0.689	0.0535	0.686	0.0539
2	R	0.584	0.0581	0.622	0.0569	0.609	0.0574
3	MI, EI	0.656	0.0567	0.671	0.0556	0.664	0.0555
4	L, *a*, *b*	0.630	0.0566	0.660	0.0553	0.663	0.0550
5	L, *a*	0.650	0.0563	0.682	0.0541	0.691	0.0542
6	L, *b*	0.657	0.0554	0.679	0.0541	0.676	0.0545
Buttock							
7	MI	0.643	0.0568	0.657	0.0553	0.665	0.0556
8	R	0.566	0.0582	0.612	0.0569	0.608	0.0577
9	MI, EI	0.636	0.0567	0.652	0.0555	0.664	0.0555
10	L, *a*, *b*	0.536	0.0590	0.578	0.0579	0.576	0.0583
11	L, *a*	0.545	0.0588	0.600	0.0572	0.586	0.0578

I—models based on spectrophotometric parameters examined by Sitek et al. [10]; II—models based on spectrophotometric parameters examined by Sitek et al. extended with the number of sunburns, exposure to UV radiation related to the longest-held occupation, and usage of photoprotectors; III—models based on spectrophotometric parameters examined by Sitek et al. extended with the number of sunburns; MI—melanin index; R—red; EI—erythema index; AUC—area under curve; SE—standard error AUC.

**Table 3:** The comparison of the quality of analyzed models validated on testing group.

Models	Spectrophotometric Variables in Models	AUC _I_ vs. AUC _II_	AUC _I_ vs. AUC _III_	AUC _II_ vs. AUC _III_
		*p*	*p*	*p*
Arm				
1	MI	0.4021	0.3095	0.7940
2	R	0.1120	0.0611	0.4129
3	MI, EI	0.4299	0.8836	0.8664
4	L, *a*, *b*	0.2965	0.1172	0.8630
5	L, *a*	0.1513	**0.0160**	0.5434
6	L, *b*	0.3147	0.1541	0.8653
Buttock				
7	MI	0.4482	0.0370	0.5872
8	R	0.0598	**0.0166**	0.7601
9	MI, EI	0.3678	0.0212	0.4377
10	L, *a*, *b*	0.1529	0.0210	0.9370
11	L, *a*	0.0427	**0.0083**	0.4839

AUC_I_—AUC for models based on spectrophotometric parameters examined by Sitek et al. [10]; AUC_II_—AUC for spectrophotometric models extended with the number of sunburns, exposure to UV radiation related to the longest-held occupation, and usage of photoprotectors; AUC_III_—AUC for spectrophotometric models extended with the number of sunburns; MI—melanin index; R—red; EI—erythema index *p*—probability for z test testing “0” hypothesis AUC_n_ = AUC_m_ vs. alternative hypothesis AUC_n_ ≠ AUC_m_. Significant differences after usage of Holm–Bonferroni correction are bolded. *p* < 0.05—result statistically significant.

**Table 4:** Comparison of the predictive quality of models to assess the probability of skin cancer occurrence according to a testing sample and on the spectrophotometric models extended by the number of sunburns (testing sample).

Compared Models		Z	*p*
MI arm	R arm	2.77	0.0055
	MI, EI arm	0.78	0.9151
	L, *a*, *b* arm	1.35	0.8289
	L, *a* arm	1.11	0.2660
	L, *b* arm	0.42	0.6747
	MI buttock	0.67	0.5043
	R buttock	2.11	0.0352
	MI, EI buttock	0.80	0.4258
	L, *a*, *b* buttock	2.50	0.0126
	L, *a* buttock	2.39	0.0170
Compared models		Z	*p*
L, *a* arm, number of sunburns	MI arm, number of sunburns	0.28	0.7819
	R arm, number of sunburns	2.61	0.0090
	MI, EI arm, number of sunburns	0.64	0.5251
	L, *a*, *b* arm, number of sunburns	0.88	0.3800
	L, *b* arm, number of sunburns	0.35	0.7279
	MI buttock, number of sunburns	0.56	0.5583
	R buttock, number of sunburns	1.62	0.1058
	MI, EI buttock, number of sunburns	0.64	0.5251
	L, *a*, *b* buttock, number of sunburns	2.10	0.0354
	L, *a* buttock, number of sunburns	1.99	0.0463

Z—The Hanley proposed algorithm for the Z model; *p*—probability of differences between compared models. The Holm–Bonferroni correction is included.

**Table 5:** Comparison of the predictive quality of spectrophotometric models extended by the number of sunburns (based on a testing sample).

Compared Models		Z	*p*
L, *a* arm, number of sunburns	MI arm, number of sunburns	0.28	0.7819
	R arm, number of sunburns	2.61	0.0090
	MI, EI arm, number of sunburns	0.64	0.5251
	L, *a*, *b* arm, number of sunburns	0.88	0.3800
	L, *b* arm, number of sunburns	0.35	0.7279
	MI buttock, number of sunburns	0.56	0.5583
	R buttock, number of sunburns	1.62	0.1058
	MI, EI buttock, number of sunburns	0.64	0.5251
	L, *a*, *b* buttock, number of sunburns	2.10	0.0354
	L, *a* buttock, number of sunburns	1.99	0.0463

Z—The Hanley proposed algorithm for the Z model; *p*—probability of differences between compared models. The Holm–Bonferroni correction is included.

## Data Availability

Not applicable.

## Supplementary Materials

The following supporting information can be downloaded at https://www.mdpi.com/article/10.3390/jcm11112969/s1, Table S1: Parameters of predictive models for assessing the probability of skin cancer occurrence based on spectrophotometric variables reported by Sitek et al. 2016 (generated on the basis of the training sample); Table S2: Parameters of spectrophotometric predictive models to assess the likelihood of skin cancers extended by environmental/behavioral factors (generated on the basis of a training sample); Table S3: Parameters of spectrophotometric predictive models for assessing the likelihood of skin cancer occurrence extended by the number of sunburns (generated on the basis of a training sample); Table S4: Comparison of the predictive quality of spectrophotometric models extended by environmental/behavioral factors (based on a testing sample); Table S5: Comparison of the predictive quality of spectrophotometric models extended by the number of sunburns (based on a testing sample).

## References

[1] Choquet H., Ashrafzadeh S., Kim Y., Asgari M.M., Jorgenson E. (2020). Genetic and environmental factors underlying keratinocyte carcinoma risk. JCI Insight.

[2] Nagarajan P., Asgari M.M., Green A.C., Guhan S.M., Arron S.T., Proby C.M., Rollison D.E., Harwood C.A., Toland A.E. (2019). Keratinocyte carcinomas: Current concepts and future research priorities. Clin. Cancer Res..

[3] Nehal K.S., Bichakjian C.K. (2018). Update on keratinocyte carcinomas. N. Engl. J. Med..

[4] Fijałkowska M., Koziej M., Antoszewski B. (2021). Detailed head localization and incidence of skin cancers. Sci. Rep..

[5] AlSalman S.A., Alkaff T.M., Alzaid T., Binamer Y. (2018). Nonmelanoma skin cancer in Saudi Arabia: Single center experience. Ann. Saudi Med..

[6] Husein-Elahmed H., Gutierrez-Salmeron M.T., Aneiros-Cachaza J., Naranjo-Sintes R. (2017). Basal cell carcinoma arising in outdoor workers versus indoor workers: A retrospective study. Cutis.

[7] Lowenstein S.E., Garrett G., Toland A.E., Jambusaria-Pahlajani A., Asgari M.M., Green A., Engels E.A., Arron S.T. (2017). Risk prediction tools for keratinocyte carcinoma after solid organ transplantation: A review of the literature. Br. J. Dermatol..

[8] Kaiser I., Pfahlberg A.B., Uter W., Heppt M.V., Veierød M.B., Gefeller O. (2020). Risk prediction models for melanoma: A systematic review on the heterogeneity in model development and validation. Int. J. Environ. Res. Public Health.

[9] Taylor S., Westerhof W., Im S., Lim J. (2006). Noninvasive techniques for the evaluation of skin color. J. Am. Acad. Dermatol..

[10] Sitek A., Rosset I., Żądzińska E., Kasielska-Trojan A., Neskoromna-Jędrzejczak A., Antoszewski B. (2016). Skin color parameters and Fitzpatrick phototypes in estimating the risk of skin cancer: A case-control study in the Polish population. J. Am. Acad. Dermatol..

[11] Hanley J.A., Hajian-Tilaki K.O. (1997). Sampling variability of nonparametric estimates of the areas under receiver operating characteristic curves: An update. Acad. Radiol..

[12] Duarte A.F., Sousa-Pinto B., Freitas A., Delgado L., Costa-Pereira A., Correia O. (2018). Skin cancer healthcare impact: A nation-wide assessment of an administrative database. Cancer Epidemiol..

[13] Linos E., Katz K.A., Colditz G.A. (2016). Skin cancer: The importance of prevention. JAMA Int. Med..

[14] Bhalla S., Kaur H., Dhall A., Raghava G.P.S. (2019). Prediction and analysis of skin cancer progression using genomics profiles of patients. Sci. Rep..

[15] Fijałkowska M., Kowalski M., Koziej M., Antoszewski B. (2021). Elevated serum levels of cathelicidin and β-defensin 2 are associated with basal cell carcinoma. Cent. Eur. J. Immunol..

[16] Roffman D., Hart G., Girardi M., Ko C.h.J., Deng J. (2018). Predicting non-melanoma skin cancer via a multi-parameterized artificial neural network. Sci. Rep..

[17] Wang H.H., Wang Y.H., Liang C.W., Li Y.C. (2019). Assessment of deep learning using nonimaging information and sequential medical records to develop a prediction model for non-melanoma skin cancer. JAMA Dermatol..

[18] Alfayez A.A., Kunz H., Lai A.G. (2021). Predicting the risk of cancer in adults using supervised machine learning: A scoping review. BMJ Open.

[19] Dessinioti C., Tzannis K., Sypsa V., Nikolaou V., Kypreou K., Antoniou C., Katsambas A., Stratigos A.J. (2011). Epidemiologic risk factors of basal cell carcinoma development and age at onset in a Southern European population from Greece. Exp. Dermatol..

[20] Khalesi M., Whiteman D.C., Tran B., Kimlin M.G., Olsen C.M., Neale R.E. (2013). A meta-analysis of pigmentary characteristics, sun sensitivity, freckling and melanocytic nevi and risk of basal cell carcinoma of the skin. Cancer Epidemiol..

