# Supplementary material for: Assessment of the Predictive Value of Spectrophotometric Skin Color Parameters and Environmental and Behavioral Factors in Estimating the Risk of Skin Cancer: A Case–Control Study
Source: J Clin Med. 2022 May 25;11(11):2969. doi: 10.3390/jcm11112969 (PMC9181677; doi:10.3390/jcm11112969)
Supplement: Supplementary file 1 [file jcm-11-02969-s001.zip › jcm-1698905-supplementary.pdf]

Table S1. Parameters of predictive models for assessing the probability of skin cancer occurrence based on spectrophotometric variables reported by Sitek et al. 2016 (generated on the basis of the training sample).

Models	Variables	Logistic regression model parameters			Skin cancer odds ratio *		
		Estimate	Walds statistics	p	OR	-95%	+95%
Arm							
1	Intercept	5.7373	7.93	**<0.0001**			
	MI	-0.2154	-0.14	**<0.0001**	0.81	0.74	0.87
2	Intercept	-9.2312	-5.79	**<0.0001**			
	R	0.0666	0.09	**<0.0001**	1.07	1.04	1.10
3	Intercept	5.3804	22.25	**<0.0001**			
	MI	-0.2469	28.42	**<0.0001**	0.78	0.71	0.86
	EI	0.1271	2.45	0.1176	1.14	0.97	1.33
4	Intercept	-11.2103	26.78	**<0.0001**			
	L	0.1660	21.36	**<0.0001**	1.18	1.10	1.27
	a	0.1364	5.17	**0.0229**	1.15	1.02	1.29
	b	0.0986	5.67	**0.0173**	1.10	1.02	1.20
5	Intercept	-10.5176	24.84	**<0.0001**			
	L	0.1809	26.43	**<0.0001**	1.20	1.12	1.28
	a	0.1490	6.27	**0.0123**	1.16	1.03	1.30
6	Intercept	-7.9351	26.73	**<0.0001**			
	L	0.1338	17.38	**<0.0001**	1.14	1.07	1.22
	b	0.1070	6.83	**0.0090**	1.11	1.03	1.21
Buttock							
7	Intercept	4.2000	18.01	**<0.0001**			
	MI	-0.1727	19.53	**<0.0001**	0.84	0.78	0.91
8	Intercept	-7.6262	19.92	**<0.0001**			
	R	0.0522	19.26	**<0.0001**	1.05	1.03	1.08
9	Intercept	3.8909	14.55	**0.0001**			
	MI	-0.1909	20.40	**0.0000**	0.83	0.76	0.90
	EI	0.0725	1.48	0.2243	1.08	0.96	1.21
10	Intercept	-8.9240	20.02	**<0.0001**			
	L	0.1406	17.32	**<0.0001**	1.15	1.08	1.23
	a	0.1185	7.98	**0.0047**	1.13	1.04	1.22
	b	0.0146	0.13	0.7201	1.01	0.94	1.10
11	Intercept	-8.8278	19.93	**<0.0001**			
	L	0.1434	19.01	**<0.0001**	1.15	1.08	1.23
	a	0.1166	7.85	**0.0051**	1.12	1.04	1.22

* The odds ratio for a unit change in individual parameters

Table S2. Parameters of spectrophotometric predictive models to assess the likelihood of skin cancers extended by environmental/behavioral factors (generated on the basis of a training sample)

Models	Variables	Logistic regression model parameters			Skin cancer odds ratio *		
		Estimate	Walds statistics	p	OR	Estimate	Walds statistics
Arm							
1	Intercept	4.8808	17.70	**0.0000**			
	MI	-0.1981	22.52	**0.0000**	0.82	0.76	0.89
	Exposure - Occupation[1]	0.0940	0.77	0.3813	1.10	0.89	1.36
	Number of sunburns[2]	0.0657	7.36	**0.0067**	1.07	1.02	1.12
	Photoprotectors - never[3]	0.1568	1.39	0.2383	1.37	0.81	2.30
2	Intercept	-8.8290	23.94	**0.0000**			
	R	0.0609	21.38	**0.0000**	1.06	1.04	1.09
	Exposure - Occupation[1]	0.0925	0.75	0.3869	1.10	0.89	1.35
	Number of sunburns[2]	0.0659	7.44	**0.0064**	1.07	1.02	1.12
	Photoprotectors - never[3]	0.1612	1.48	0.2238	1.38	0.82	2.32
3	Intercept	4.6458	15.47	**0.0001**			
	MI	-0.2205	21.70	**0.0000**	0.80	0.73	0.88
	EI	0.0890	1.14	0.2856	1.09	0.93	1.29
	Exposure - Occupation[1]	0.0984	0.84	0.3602	1.10	0.89	1.36
	Number of sunburns[2]	0.0623	6.66	**0.0099**	1.06	1.02	1.12
	Photoprotectors - never[3]	0.1512	1.28	0.2570	1.35	0.80	2.28
4	Intercept	-10.3544	21.72	**0.0000**			
	L	0.1470	15.97	**0.0001**	1.16	1.08	1.24
	a	0.0955	2.40	0.1210	1.10	0.98	1.24
	b	0.1094	6.67	**0.0098**	1.12	1.03	1.21
	Exposure - Occupation[1]	0.1134	1.10	0.2941	1.12	0.91	1.38
	Number of sunburns[2]	0.0654	7.16	**0.0074**	1.07	1.02	1.12
	Photoprotectors - never[3]	0.1808	1.81	0.1784	1.44	0.85	2.43
5	Intercept	-9.5614	19.63	**0.0000**			
	L	0.1634	20.66	**0.0000**	1.18	1.10	1.26
	a	0.1107	3.29	0.0695	1.12	0.99	1.26
	Exposure - Occupation[1]	0.0976	0.83	0.3613	1.10	0.89	1.36
	Number of sunburns[2]	0.0642	7.01	**0.0081**	1.07	1.02	1.12

	Photoprotectors - never[3]	0.1628	1.51	0.2188	1.38	0.82	2.33
6	Intercept	-8.0668	25.26	**0.0000**			
	L	0.1242	14.03	**0.0002**	1.13	1.06	1.21
	b	0.1155	7.56	**0.0060**	1.12	1.03	1.22
	Exposure - Occupation[1]	0.1087	1.02	0.3128	1.11	0.90	1.38
	Number of sunburns[2]	0.0717	8.46	**0.0036**	1.07	1.02	1.13
	Photoprotectors - never[3]	0.2004	2.26	0.1327	1.49	0.89	2.52
Buttock							
7	Intercept	3.4498	11.55	**0.0007**			
	MI	-0.1612	16.17	**0.0001**	0.85	0.79	0.92
	Exposure - Occupation[1]	0.1349	1.59	0.2068	1.14	0.93	1.41
	Number of sunburns[2]	0.0687	7.57	**0.0059**	1.07	1.02	1.12
	Photoprotectors - never[3]	0.1966	2.28	0.1312	1.48	0.89	2.47
8	Intercept	-7.5248	18.10	**0.0000**			
	R	0.0484	15.76	**0.0001**	1.05	1.02	1.07
	Exposure - Occupation[1]	0.1299	1.49	0.2223	1.14	0.92	1.40
	Number of sunburns[2]	0.0689	7.65	**0.0057**	1.07	1.02	1.12
	Photoprotectors - never[3]	0.1965	2.28	0.1311	1.48	0.89	2.47
9	Intercept	3.3364	10.28	**0.0013**			
	MI	-0.1690	15.22	**0.0001**	0.84	0.78	0.92
	EI	0.0302	0.24	0.6258	1.03	0.91	1.16
	Exposure - Occupation[1]	0.1333	1.55	0.2125	1.14	0.93	1.41
	Number of sunburns[2]	0.0666	7.04	**0.0080**	1.07	1.02	1.12
	Photoprotectors - never[3]	0.1928	2.18	0.1397	1.47	0.88	2.45
10	Intercept	-8.3446	16.64	**0.0000**			
	L	0.1231	12.78	**0.0004**	1.13	1.06	1.21
	a	0.0855	3.91	0.0479	1.09	1.00	1.19
	b	0.0387	0.83	0.3609	1.04	0.96	1.13
	Exposure - Occupation[1]	0.1388	1.66	0.1975	1.15	0.93	1.42
	Number of sunburns[2]	0.0690	7.47	**0.0063**	1.07	1.02	1.13
	Photoprotectors - never[3]	0.1959	2.22	0.1364	1.48	0.88	2.48
11	Intercept	-8.0912	15.88	**0.0001**			
	L	0.1309	15.21	**0.0001**	1.14	1.07	1.22
	a	0.0813	3.56	0.0593	1.08	1.00	1.18

	Exposure - Occupation[1]	0.1274	1.43	0.2316	1.14	0.92	1.40
	Number of sunburns[2]	0.0673	7.21	**0.0072**	1.07	1.02	1.12
	Photoprotectors - never[3]	0.1853	2.01	0.1562	1.45	0.87	2.42

* The odds ratio for a unit change in individual parameters

[1] exposure to UV radiation related to the longest-performed occupation - a continuous variable expressed on a scale of 1-5 (1-no exposure, 5- very high exposure)

[2] lifetime total number of sunburns

[3] not using photoprotectors (never) vs using them ever (rarely, sometimes, often, always)

Table S3. Parameters of spectrophotometric predictive models for assessing the likelihood of skin cancer occurrence extended by the number of sunburns (generated on the basis of a training sample)

Models	Variables	Logistic regression model parameters			Skin cancer odds ratio *		
		Estimate	Walds statistics	p	OR	Estimate	Walds statistics
Arm							
1	Intercept	5.2785	21.58	**0.0000**			
	MI	-0.2066	25.04	**0.0000**	0.81	0.75	0.88
	Number of sunburns[1]	0.0685	8.55	**0.0034**	1.07	1.02	1.12
2	Intercept	-9.049	25.86	**0.0000**			
	R	0.064	23.93	**0.0000**	1.07	1.04	1.09
	Number of sunburns[1]	0.069	8.61	**0.0034**	1.07	1.02	1.12
3	Intercept	3.5797	12.13	**0.0005**			
	MI	-0.1732	16.59	**0.0000**	0.84	0.77	0.91
	EI	0.0390	0.40	0.5259	1.04	0.92	1.17
	Number of sunburns[1]	0.0705	8.44	**0.0037**	1.07	1.02	1.13
4	Intercept	-10.5389	23.32	**0.0000**			
	L	0.1556	18.39	**0.0000**	1.17	1.09	1.25
	a	0.1020	2.78	0.0952	1.11	0.98	1.25
	b	0.1029	6.06	**0.0138**	1.11	1.02	1.20
	Number of sunburns[1]	0.0683	8.33	**0.0039**	1.07	1.02	1.12
5	Intercept	-9.7976	21.28	**0.0000**			
	L	0.1707	23.08	**0.0000**	1.19	1.11	1.27
	a	0.1159	3.66	0.0557	1.12	1.00	1.26
	Number of sunburns[1]	0.0667	8.10	**0.0044**	1.07	1.02	1.12
6	Intercept	-8.1332	26.55	**0.0000**			
	L	0.1320	16.20	**0.0001**	1.14	1.07	1.22
	b	0.1093	6.96	**0.0084**	1.12	1.03	1.21
	Number of sunburns[1]	0.0751	9.91	**0.0016**	1.08	1.03	1.13
Buttock							
7	Intercept	3.7342	13.97	**0.0002**			
	MI	-0.1633	17.25	**0.0000**	0.85	0.79	0.92
	Number of sunburns[1]	0.0733	9.21	**0.0024**	1.08	1.03	1.13
8	Intercept	-7.4354	18.73	**0.0000**			
	R	0.0493	16.97	**0.0000**	1.05	1.03	1.08
	Number of sunburns[1]	0.0733	9.25	**0.0024**	1.08	1.03	1.13
9	Intercept	3.5797	12.13	**0.0005**			
	MI	-0.1732	16.59	**0.0000**	0.84	0.77	0.91
	EI	0.0390	0.40	0.5259	1.04	0.92	1.17

	Number of sunburns[1]	0.0705	8.44	**0.0037**	1.07	1.02	1.13
10	Intercept	-8.3554	17.54	**0.0000**			
	L	0.1294	14.60	**0.0001**	1.14	1.07	1.22
	a	0.0938	4.82	**0.0281**	1.10	1.01	1.19
	b	0.0252	0.37	0.5443	1.03	0.95	1.11
	Number of sunburns[1]	0.0719	8.68	**0.0032**	1.07	1.02	1.13
11	Intercept	-8.1948	17.12	**0.0000**			
	L	0.1344	16.62	**0.0000**	1.14	1.07	1.22
	a	0.0907	4.56	**0.0328**	1.09	1.01	1.19
	Number of sunburns[1]	0.0708	8.52	**0.0035**	1.07	1.02	1.13

* The odds ratio for a unit change in individual parameters

[1] lifetime total number of sunburns

Table S4. Comparison of the predictive quality of spectrophotometric models extended by environmental/behavioral factors (based on a testing sample)

Compared models		Z	p
MI arm, exposure - occupation, number of sunburns, photoprotectors	R arm, exposure - occupation, number of sunburns, photoprotectors	-2.57	0.0102
	MI, EI arm, exposure - occupation, number of sunburns, photoprotectors	-1.15	0.2491
	L, a, b arm, exposure - occupation, number of sunburns, photoprotectors	-1.02	0.3078
	L, a arm, exposure - occupation, number of sunburns, photoprotectors	-0.56	0.5747
	L, b arm, exposure - occupation, number of sunburns, photoprotectors	-0.30	0.7604
	MI buttock, exposure - occupation, number of sunburns, photoprotectors	-0.76	0.4493
	R buttock, ekspozycja – zawód, number of sunburns, photoprotectors	-1.74	0.0811
	MI, EI buttock, ekspozycja – zawód, number of sunburns, photoprotectors	-0.90	0.3702

The Holm-Bonferroni correction is included

Table S5. Comparison of the predictive quality of spectrophotometric models extended by the number of sunburns (based on a testing sample)

Compared models		Z	p
L, a arm, number of sunburns	MI arm, number of sunburns	0.28	0.7819
	MI, EI arm, number of sunburns	0.64	0.5251
	L, a, b arm, number of sunburns	0.88	0.3800
	L, b arm, number of sunburns	-0.35	0.7279
	MI buttock, number of sunburns	-0.56	0.5583
	MI, EI buttock, number of sunburns	-0.64	0.5251

The Holm-Bonferroni correction is included
